# RTGNet: A Dual-Branch Network Integrating Recurrent Texture and Temporal Dynamics from sEMG for Lower-Limb Joint Angle Prediction

**DOI:** 10.3390/s26165144

**Published:** 2026-08-14

**Authors:** Zhiwei Hu, Quansheng Xu, Shaowei Su, Yinggan Tang, Yonghong Xu

**Affiliations:** 1 School of Computer Science and Technology, Huaibei Normal University, Huaibei 235000, China; 12411080245@chnu.edu.cn (Z.H.); 12412080601@chnu.edu.cn (S.S.); xuyh@chnu.edu.cn (Y.X.); 2 School of Electrical Engineering, Yanshan University, Qinhuangdao 066004, China; ygtang@ysu.edu.cn

**Keywords:** surface electromyography (sEMG), recurrence plot, gated fusion, temporal dynamics, BiLSTM, joint angle prediction

## Abstract

Accurate continuous prediction of lower-limb joint angles from surface electromyography (sEMG) remains challenging because of the nonlinear, non-stationary, and subject-specific nature of sEMG signals, which can reduce robustness and lead to degraded prediction accuracy during highly dynamic gait phases. In this study, we propose RTGNet, a dual-branch deep learning framework for lower-limb joint-angle prediction from multichannel sEMG. The method constructs two feature views from the same sEMG stream: recurrence-plot (RP)-based representations for nonlinear texture characterization and time-series sequences for long-term temporal dependency modeling. These views are processed by a convolutional neural network (CNN) with a convolutional block attention module (CBAM) and a bidirectional long short-term memory network (BiLSTM), respectively, and integrated through an adaptive gated fusion mechanism. An enhanced Huber-TopK loss is further employed to emphasize samples with large prediction errors. Experiments on the SIAT-LLMD dataset under an offline cross-subject evaluation setting show that RTGNet achieves a mean absolute error (MAE) of 3.87°, a root mean square error (RMSE) of 5.25°, and an $R^{2}$ of 0.81 during walking, as well as an MAE of 4.53°, an RMSE of 6.47°, and an $R^{2}$ of 0.84 during stair ascent. The proposed framework outperforms temporal-only and RP-based baselines, and ablation results further support the effectiveness of the gated fusion strategy and CBAM attention. Overall, these results suggest that integrating recurrence texture and temporal dynamics is a promising strategy for sEMG-driven joint-angle prediction and provides a useful basis for future exoskeleton control-oriented studies.

## 1. Introduction

Accurate and continuous estimation of lower-limb joint angles is a fundamental problem in gait analysis, rehabilitation assessment, exoskeleton control, and human–machine interaction systems. In clinical and wearable applications, joint kinematics not only reflect motor function but also directly influence control strategies and motion intention decoding [1]. Traditional motion capture systems, such as Vicon-based optical tracking systems, provide high-precision measurements but are restricted to laboratory environments and are expensive. In contrast, inertial measurement units (IMUs) offer better portability but suffer from drift and magnetic disturbances. To address real-world applicability, Hernandez et al. proposed a deep learning-based framework using wearable IMU signals for lower-limb kinematic estimation during walking and running tasks [2].

Surface electromyography (sEMG) has attracted increasing attention due to its ability to directly reflect neuromuscular activation. Coker et al. proposed a model that integrates sEMG signals and historical joint angles for knee angle prediction [3], while Liang et al. introduced a synergy-based approach for knee kinematic estimation using thigh motion information [4]. These studies demonstrate a nonlinear relationship between muscle activation and joint motion.

Early sEMG-based approaches mainly relied on handcrafted features and shallow regression models. To support this line of research, Wei et al. provided a comprehensive surface electromyogram and kinematic dataset for movement intent recognition [5]. To better understand muscle dynamics, recurrence quantification analysis (RQA) was introduced to quantify the dynamical coordination among muscles [6]. In addition, muscle synergy-based representations have been widely explored to analyze muscle coordination patterns, including the effects of fatigue during running [7]. Such approaches are also capable of reducing dimensionality and improving physiological interpretability [8,9]. However, these methods rely heavily on manual feature engineering and exhibit limited generalization under inter-subject variability and complex gait conditions.

With the development of deep learning, recurrent neural networks (RNNs) and long short-term memory (LSTM) networks have been widely employed to model temporal dependencies. For instance, Gupta et al. proposed a framework for continuous angular position estimation of the human ankle during unconstrained locomotion [10].

Beyond pure recurrent architectures, hybrid deep learning models have been extensively explored to improve feature representation and prediction performance. Lu et al. proposed a stacked CNN–LSTM model for simultaneous multi-joint angle estimation by integrating convolutional feature extraction with temporal dependency modeling [11]. Zhang et al. introduced a transfer learning-based dual-branch EMG network to enhance cross-subject motion intention recognition performance [12]. Chen et al. developed a multimodal fusion network combining sEMG and force myography (FMG) signals for joint angle estimation, highlighting the benefit of multi-sensor complementary learning [13]. More recently, Sharifi-Renani et al. proposed BioMAT, a transformer-based framework for multi-activity biomechanical modeling using wearable sensors, enabling unified kinematic prediction across diverse motion tasks [14]. Han et al. proposed a temporal convolutional network (TCN)- and convolutional block attention module (CBAM)-based model for continuous lower-limb joint angle estimation from sEMG signals, achieving strong performance in multi-joint and multi-task scenarios [15]. In addition, the complexity of EMG signals has been investigated from physiological and signal-processing perspectives. Studies of muscle co-activation in the time–frequency domain reveal nonlinear coordination among multiple muscles during gait [16].

Despite these advances, most existing methods treat sEMG signals as one-dimensional time series, neglecting their nonlinear dynamical structures and inter-channel spatial relationships. Moreover, single-branch architectures struggle to capture both long-term temporal dependencies and structural representations. Time–frequency transformations, including the short-time Fourier transform and wavelet transform, have therefore been explored to enrich sEMG representations [17,18]. However, such representations do not explicitly characterize recurrence relationships between temporal states. Recurrence plots (RP), a well-established tool for analyzing nonlinear dynamical systems [6], provide a direct visualization of temporal recurrence structures. RP-based representations have been successfully applied in electroencephalography (EEG)-based seizure detection [19]. Nevertheless, their use for sEMG-driven lower-limb joint-angle prediction remains largely unexplored.

These limitations motivate RTGNet, whose main contributions are threefold. First, a novel parallel architecture integrates an RP-CNN branch for spatial texture extraction with a BiLSTM branch for temporal dynamics modeling, enabling structural–temporal feature fusion for sEMG-driven kinematics prediction. Second, an adaptive gated fusion mechanism dynamically weights the two feature views, while a peak-enhanced Huber-TopK loss emphasizes samples with large prediction errors while retaining robust regression. Third, evaluation on the public SIAT-LLMD dataset across multiple subjects and gait tasks shows that RTGNet outperforms the evaluated unimodal baselines under the present subject-wise split.

## 2. Methods

**Figure 1:**
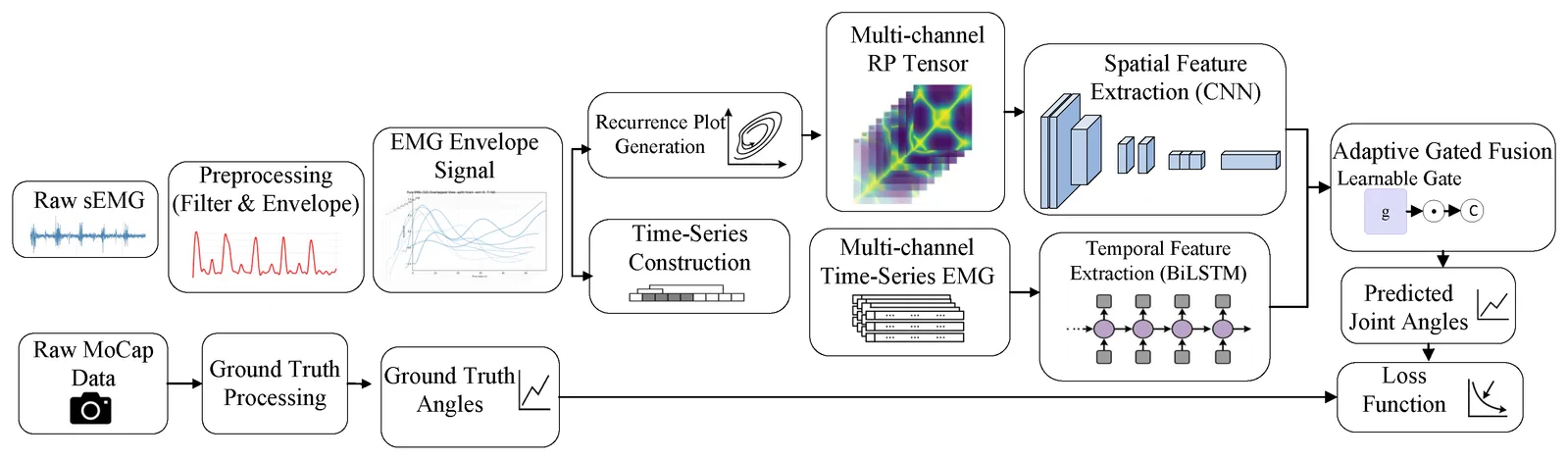
Overall framework of the proposed RTGNet for sEMG-driven lower-limb joint angle prediction.

The overall framework of the proposed RTGNet is illustrated in Figure 1. The pipeline consists of three main stages: signal preprocessing, dual-branch multimodal feature extraction (RP images and 1D temporal sequences), and adaptive gated fusion for joint angle prediction. The specific details of each module are elaborated in the following subsections.

### 2.1. Dataset

The proposed RTGNet was evaluated on the open-source SIAT-LLMD dataset [5], which contains synchronized sEMG, kinematic, and kinetic recordings from 40 healthy adults (30 males and 10 females), denoted as Sub01–Sub40. The average age of the participants was 24.5 years (range: 19–33 years), and the average body weight was 63.8 kg (range: 46.3–85 kg).

The SIAT-LLMD dataset provides biomechanical data for 16 lower-limb movement tasks. In this study, two representative tasks were selected, namely walking (WAK) and stair ascent (UPS). Nine sEMG channels were recorded from the left leg using a wireless acquisition system ( Delsys Inc., Natick, MA, USA) at 1926 Hz. The recorded muscles were the tensor fascia lata, rectus femoris, vastus medialis, semimembranosus, upper tibialis anterior, lower tibialis anterior, lateral gastrocnemius, medial gastrocnemius, and soleus. The original kinematic data were acquired using a six-camera optical motion capture system (Eagle, Motion Analysis Corporation, Rohnert Park, CA, USA) with 41 cutaneous reflective markers. The marker trajectories were processed using the OpenSim Gait 2392 model to calculate joint kinematics. The released comma-separated values (CSV) files provide synchronized sEMG, kinematic, and kinetic recordings on a shared timeline. In this study, the bilateral hip, knee, and ankle flexion angles in the kinematic recordings were used as the reference ground-truth targets for model training and accuracy evaluation.

### 2.2. Signal Preprocessing

**Figure 2:**
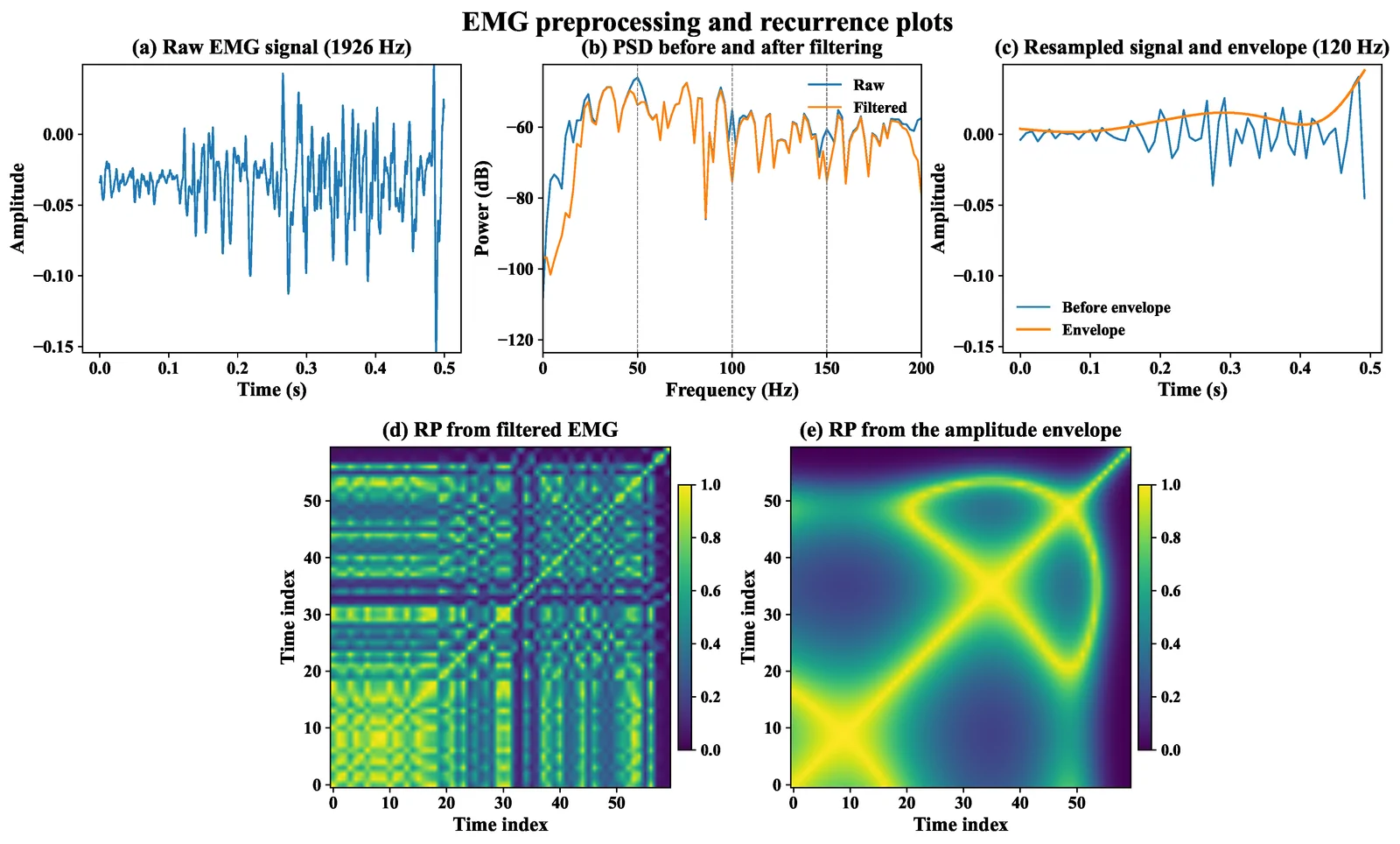
Overview of the zero-phase sEMG preprocessing, temporal-resolution unification, and channel-wise recurrence-plot generation pipeline.

Figure 2 summarizes the preprocessing and RP construction pipeline. Figure 2a shows the raw sEMG signal, whereas Figure 2b compares its power spectral density before and after filtering. The signal was processed using a fourth-order Butterworth band-pass filter at 20–450 Hz, a 50-Hz notch filter, and additional notch filters at its harmonic frequencies. It was then full-wave rectified and low-pass filtered at 6 Hz. The resulting resampled signal and linear envelope are shown in Figure 2c. The envelope represents the slowly varying muscle-activation intensity and preserves the temporal trend relevant to joint-angle prediction while suppressing rapid oscillations. Figure 2d,e show the RPs constructed from the filtered EMG and its amplitude envelope, respectively. The joint-angle trajectories were processed using a fourth-order low-pass filter with an 8 Hz cutoff frequency. All filters were implemented using forward–backward filtering with the filtfilt function from SciPy version 1.13.1 to avoid phase distortion.

The resampled sEMG envelopes and joint-angle trajectories were aligned on a common timeline. No explicit electromechanical-delay compensation was introduced; the temporal relationship between muscle activation and joint motion was learned from the input sequence. Since forward–backward filtering uses samples from both temporal directions, the present preprocessing procedure is applicable to the offline evaluation setting considered in this study, whereas causal filtering would be required for real-time deployment.

#### Subject-Wise Data Partitioning and Window Generation

For cross-subject evaluation, the 40 participants were assigned to fixed training (n=28), validation (n=6), and test (n=6) subsets using a random seed of 42. This was a fixed subject-wise split rather than a k-fold cross-validation procedure. Subject assignment was completed before sliding-window generation, and each participant contributed data to a single subset. Feature-standardization parameters were estimated from the training subset and applied to the validation and test subsets.

Sliding windows were subsequently constructed within each subset. Each input window contained 60 samples (0.5 s at 120 Hz), and the joint-angle vector at the immediately subsequent sample was used as the prediction target. The training and validation subsets used a stride of 30 samples, while the test subset used a stride of 1 sample for dense trajectory reconstruction.

**Figure 3:**
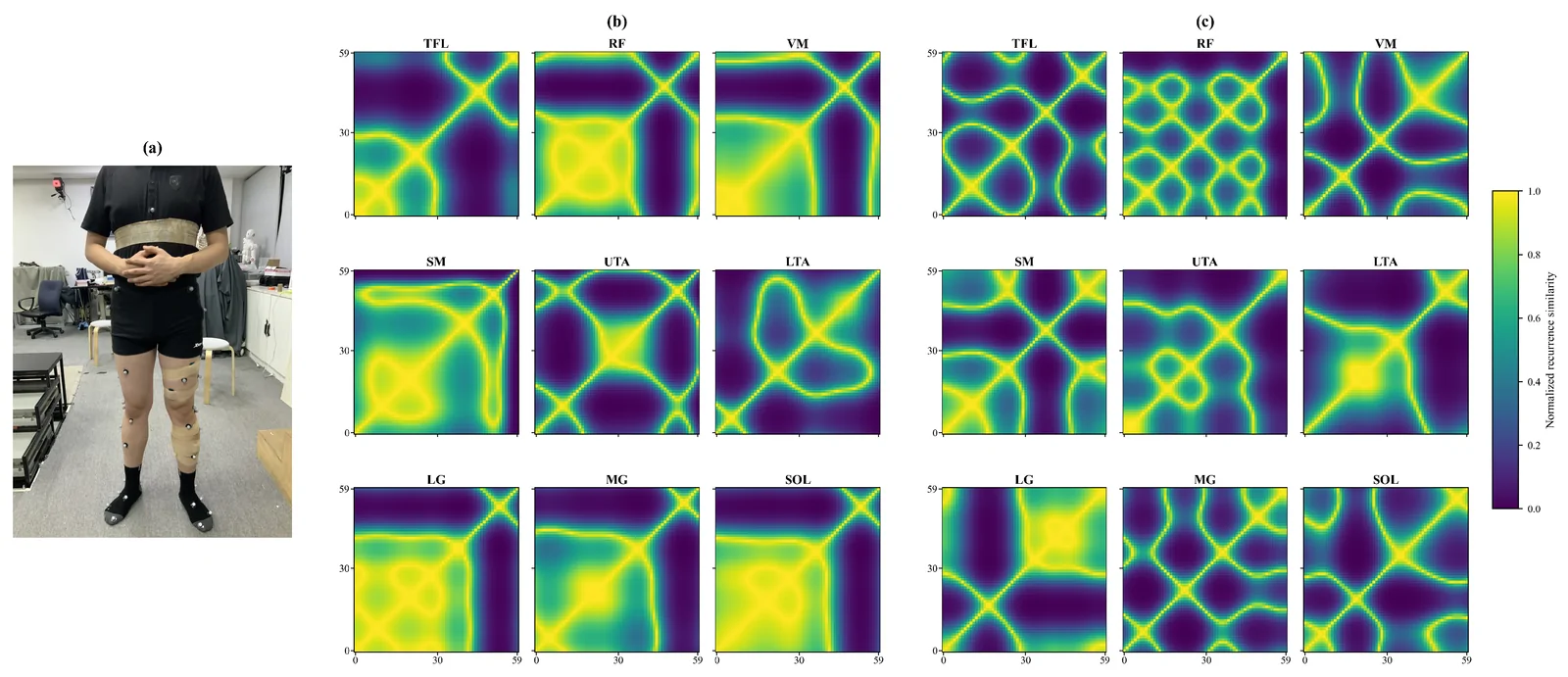
Representative sensor and marker placement together with channel-wise recurrence-plot (RP) examples. (**a**) Representative placement of sEMG sensors and reflective markers. The nine sEMG channels were recorded from the left tensor fascia lata (TFL), rectus femoris (RF), vastus medialis (VM), semimembranosus (SM), upper tibialis anterior (UTA), lower tibialis anterior (LTA), lateral gastrocnemius (LG), medial gastrocnemius (MG), and soleus (SOL). (**b**,**c**) Channel-wise RPs for a fixed held-out subject (Subject 16) during WAK and UPS, respectively. All RPs were generated from 60-sample input windows using the same preprocessing and normalization procedure.

The sensor and marker placement and the representative channel-wise RP examples used in the dataset are shown in Figure 3.

Given that sEMG signals exhibit pronounced nonlinear and non-stationary characteristics, relying solely on 1D time-domain waveforms makes it difficult to fully characterize their evolving structural patterns. The continuous recurrence plot (RP) maps each 1D channel within a temporal window to a 2D time–time similarity matrix. For an sEMG window ${\left\{x_{t}^{\left(d\right)}\right\}}_{t=1}^{T}$ of channel *d*, with T=60, channel-wise standardization is first performe d:

(1) $${\tilde{x}}_{t}^{\left(d\right)}=\frac{x_{t}^{\left(d\right)}-\mu_{x}^{\left(d\right)}}{\sigma_{x}^{\left(d\right)}+\varepsilon },\;t=1,\ldots ,T$$

where $\mu_{x}^{\left(d\right)}$ and $\sigma_{x}^{\left(d\right)}$ are respectively the mean and sample standard deviation of the *T* values in the current window, and $\varepsilon =10^{-6}$ prevents division by zero. The pairwise absolute-distance matrix $D^{\left(d\right)}\in R^{T\times T}$ is then constructed as

(2) $$d_{ij}^{\left(d\right)}=\left|{\tilde{x}}_{i}^{\left(d\right)}-{\tilde{x}}_{j}^{\left(d\right)}\right|,\;i,j=1,\ldots ,T$$

The adaptive scale parameter was defined as the sample standard deviation of all $T^{2}$ elements in the distance matrix:

(3) $${\bar{d}}^{\left(d\right)}=\frac{1}{T^{2}}\sum_{i=1}^{T}\sum_{j=1}^{T}d_{ij}^{\left(d\right)},\;s^{\left(d\right)}=\sqrt{\frac{1}{T^{2}-1}\sum_{i=1}^{T}\sum_{j=1}^{T}{\left(d_{ij}^{\left(d\right)}-{\bar{d}}^{\left(d\right)}\right)}^{2}}+\varepsilon \in R$$

Here, *i* and *j* independently range from 1 to *T*, and $s^{\left(d\right)}$ is a scalar shared by all elements of the corresponding recurrence matrix. The exponential-kernel similarity matrix is defined as

(4) $$K_{ij}^{\left(d\right)}=\exp\left(-\frac{d_{ij}^{\left(d\right)}}{s^{\left(d\right)}}\right),\;i,j=1,\ldots ,T$$

The resulting matrix is subsequently normalized to the range [0,1]:

(5) $$R_{ij}^{\left(d\right)}=\frac{K_{ij}^{\left(d\right)}-K_{\min}^{\left(d\right)}}{K_{\max}^{\left(d\right)}-K_{\min}^{\left(d\right)}+\varepsilon },\;R^{\left(d\right)}\in {\left[0,1\right]}^{T\times T}$$

where $K_{\min}^{\left(d\right)}=\min_{i,j}K_{ij}^{\left(d\right)}$ and $K_{\max}^{\left(d\right)}=\max_{i,j}K_{ij}^{\left(d\right)}$. A value closer to 1 denotes greater similarity between the states at two time points. The nine channel-specific RP matrices are stacked along the channel dimension to form the 9×T×T tensor supplied to the RP-CNN branch.

### 2.3. RTGNet Architecture

**Figure 4:**
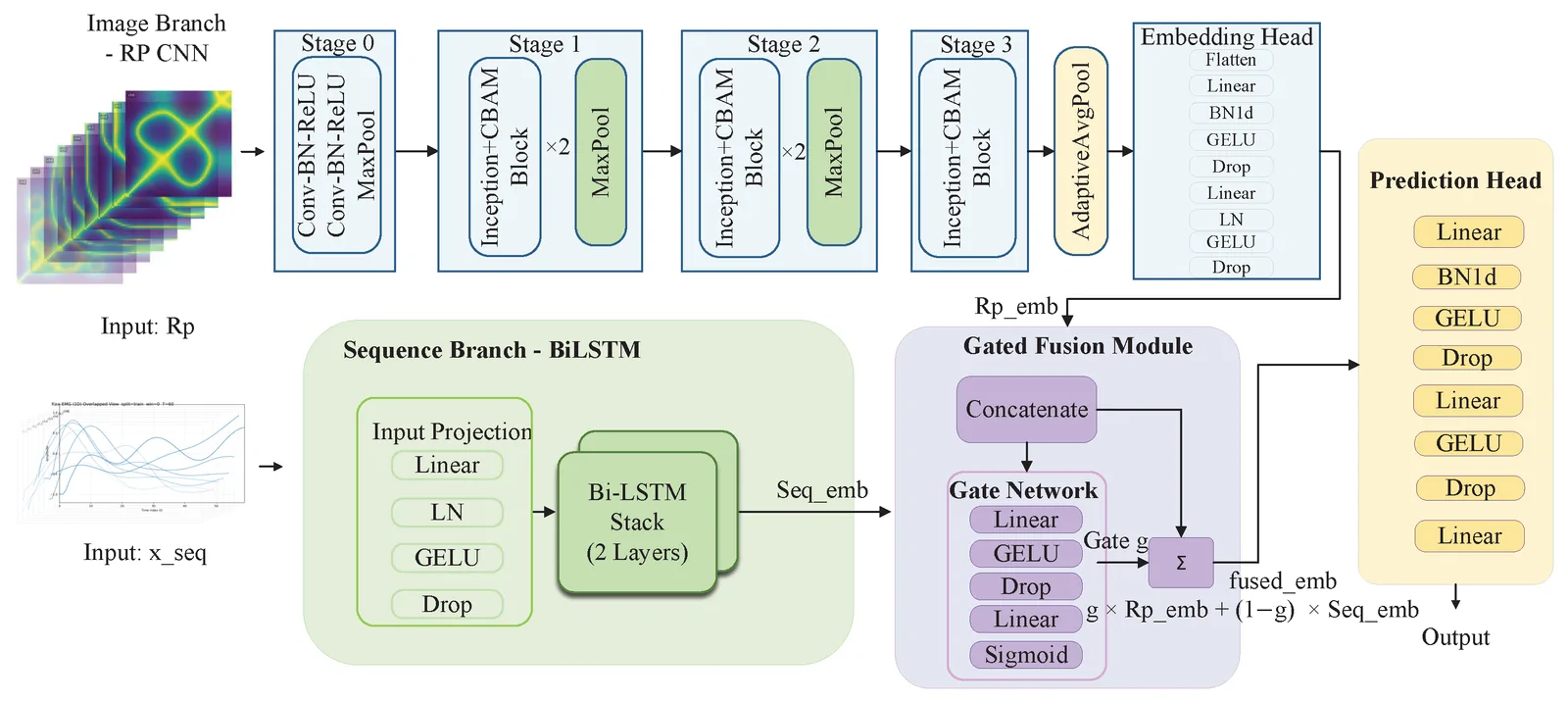
Detailed architecture of the multimodal RTGNet, integrating the RP-CNN spatial branch and the BiLSTM temporal branch.

To effectively capture both the complex neuromuscular mapping and the temporal evolution of joint kinematics, we propose the Recurrence-Temporal Gated Network (RTGNet). As illustrated in Figure 4, this framework consists of two parallel feature extraction branches followed by an adaptive gated fusion module.

#### 2.3.1. RP-CNN Branch for Spatial Textures

**Figure 5:**
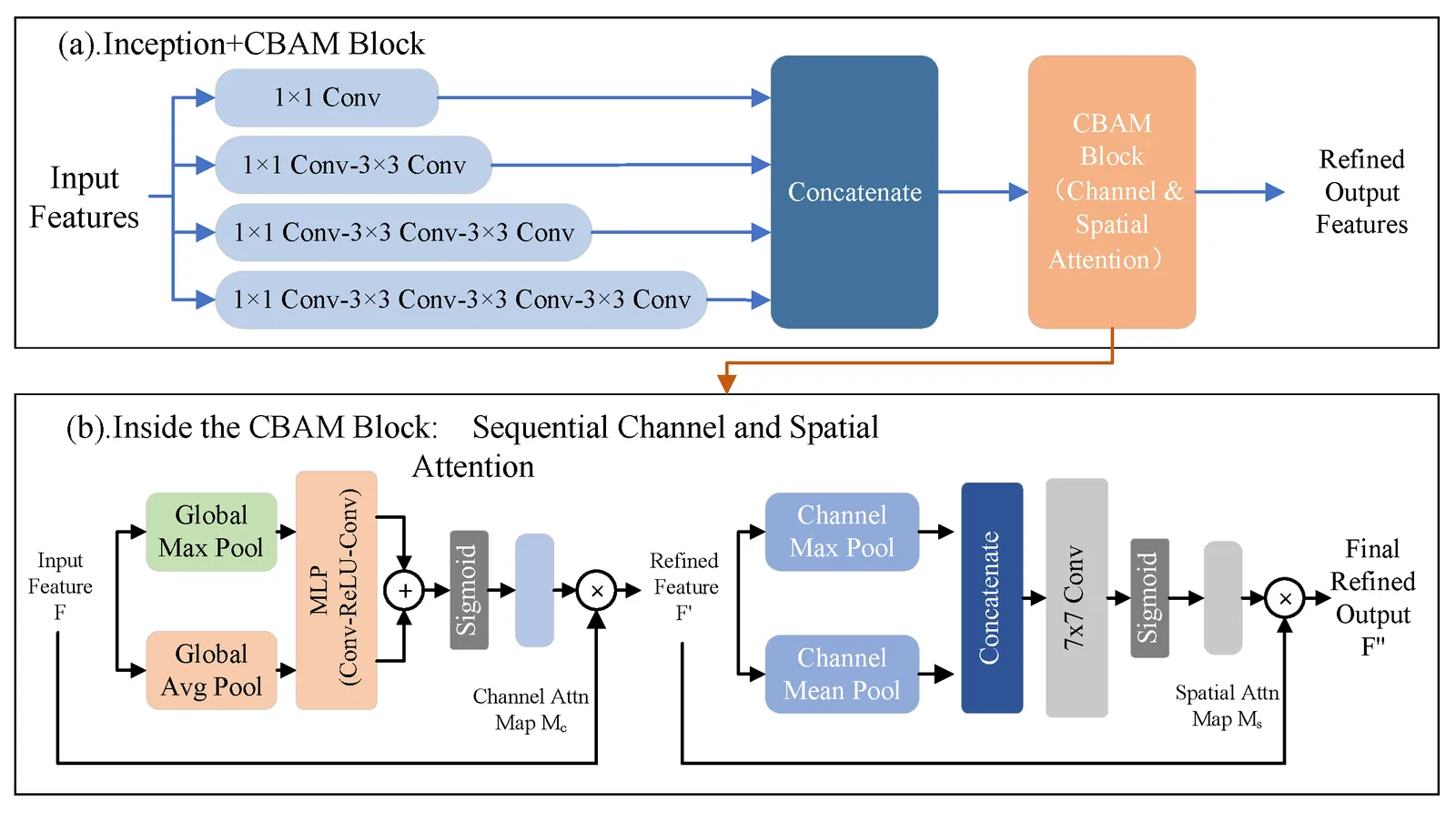
Structure of the Inception block with the Convolutional Block Attention Module (CBAM). (**a**) Multi-scale convolutional pathways and feature concatenation followed by CBAM refinement. (**b**) Sequential channel- and spatial-attention operations inside CBAM.

The RP-CNN branch extracts high-level spatial texture features from the 2D recurrence plots. The input is a multi-channel RP tensor $X_{rp}\in R^{B\times C\times H\times W}$, where *B* is the batch size, *C* is the number of sEMG channels, and H×W denotes the resolution of the RP. As shown in Figure 5a, parallel convolutional pathways extract multi-scale textures before feature concatenation. The concatenated features are refined by the convolutional block attention module (CBAM) [20], whose channel- and spatial-attention operations are illustrated in Figure 5b. This refinement suppresses less informative responses and emphasizes recurrence-texture regions that may contain information relevant to joint-angle prediction. Adaptive average pooling is finally applied to generate a fixed-dimensional global spatial embedding $h_{rp}$.

#### 2.3.2. BiLSTM Branch for Temporal Dynamics

Parallel to the RP-CNN, the BiLSTM branch models forward and backward temporal dependencies. The input is a 1D time-series tensor $X_{seq}\in R^{B\times T\times C}$, where T=60 represents the sliding-window length. A two-layer bidirectional LSTM encodes the dynamic context. Average pooling is applied along the time dimension, followed by a projection block (Linear–LayerNorm–GELU–Dropout) to compress the feature into a 256-dimensional temporal embedding $h_{seq}$.

#### 2.3.3. Adaptive Gated Fusion

To synergistically integrate the complementary modalities, a gated fusion mechanism is introduced. Let *D* be the embedding dimension (D=256). The fusion gate weight $g\in R^{D}$ is generated by concatenating $h_{rp}$ and $h_{seq}$:

(6) $$g=\sigma \left(W_{g}\left[h_{rp};h_{seq}\right]+b_{g}\right)$$

where $W_{g}$ and $b_{g}$ denote the learnable weight matrix and bias vector, respectively, [·;·] denotes concatenation, and σ is the sigmoid activation. The fused multimodal representation $h_{fused}$ is obtained via a soft-gating mechanism:

(7) $$h_{fused}=g⊙h_{rp}+\left(1-g\right)⊙h_{seq}$$

where ⊙ denotes element-wise multiplication. Finally, $h_{fused}$ is fed into the prediction head to output the continuous multi-joint angles.

#### 2.3.4. Analysis of Cross-Branch Representations

The 256-dimensional RP-CNN and BiLSTM embeddings, denoted by $h_{rp}$ and $h_{seq}$, were extracted before gated fusion. Cross-branch Pearson correlations were calculated across test samples, and their mean absolute value was used to summarize feature-wise similarity. Linear centered kernel alignment (CKA) was calculated as a global measure of representation similarity. The two embedding sets were also projected into two dimensions using t-distributed stochastic neighbor embedding (t-SNE) with a fixed random seed. The analysis used the same multimodal checkpoint and test windows as the main evaluation.

### 2.4. Peak-Enhanced Huber-TopK Loss

A critical challenge in joint-angle regression is that samples with large prediction errors can be underweighted when the loss is averaged across the full gait cycle. Such samples may occur during rapid flexion or extension phases, but they are not restricted to those phases.

To address the issue above, we propose a Peak-Enhanced Huber-TopK loss. First, we compute the standard channel-averaged Huber loss for each sample in a mini-batch of size *B*, yielding the overall mean error as the base loss ($L_{base}$).

To emphasize hard samples, which may include samples with large deviations during rapid transitions or at extreme joint angles, we dynamically sort the individual sample errors in descending order. We then select the top ρ proportion of samples with the highest errors to form a Top-K set, where the number of selected samples is $N_{\mathrm{topK}}=\max\left(1,\left\lfloor B\rho \right\rfloor \right)$. A penalty term for these high-error samples is added to the base loss to formulate the final total loss:

(8) $$L_{total}=L_{base}+\lambda_{\mathrm{topK}}\frac{1}{N_{\mathrm{topK}}}\sum_{j=1}^{N_{\mathrm{topK}}}e_{\left(j\right)}$$

where $e_{\left(j\right)}$ represents the error of the *j*-th sample in the sorted Top-K set, and $\lambda_{\mathrm{topK}}$ is the weighting coefficient for the enhancement term.

The Huber transition parameter was set to δ=1.0 for targets normalized to [0,1]. The Top-K ratio and enhancement weight were selected on the WAK validation set. All remaining training settings, including B=128, the random seed, optimizer, learning-rate schedule, and early-stopping procedure, were held constant. The candidate values were ρ∈{0.01,0.05,0.10} and $\lambda_{\mathrm{topK}}\in \left\{1,3,5\right\}$. The parameter combination with the lowest validation MAE was selected. Table 1 shows that ρ=0.05 and $\lambda_{\mathrm{topK}}=3$ yielded the lowest validation MAE. The final loss therefore used δ=1.0, ρ=0.05, and $\lambda_{\mathrm{topK}}=3$, corresponding to $N_{\mathrm{topK}}=6$ samples in a full mini-batch.

## 3. Results

### 3.1. Experimental Setup

RTGNet was implemented in PyTorch version 2.7.0 and trained on an NVIDIA GeForce RTX 5060 GPU. The random seed was fixed to 42, and deterministic computation was enabled for reproducibility. The RP input was represented as (B,C,60,60), and the temporal input was represented as (B,60,C), where C=9 denotes the sEMG channels.

RTGNet was trained for up to 500 epochs with an early-stopping patience of 100. AdamW was used with a batch size of 128, an initial learning rate of $1\times 10^{-3}$, and a weight decay of $1\times 10^{-4}$. A cosine annealing scheduler with a 5-epoch warm-up was applied, and the minimum learning rate was set to 1% of the initial value. The Peak-Enhanced Huber-TopK loss used δ=1.0, ρ=0.05, and $\lambda_{\mathrm{topK}}=3$.

For comparison, temporal baselines used only sequential sEMG inputs, while RP-based baselines used only RP images. Recent lightweight RP backbones, including EfficientViT-M0, RepViT-M0.9, and FastViT-T8, were trained under the same RP-only setting. Their RP images were resized from 60×60 to 224×224 by bilinear interpolation to match the standard input size of these image backbones.

Pearson’s correlation coefficient (CC) was additionally computed to assess waveform similarity. For each test subject *s* and joint *j*, CC was calculated over all dense test windows as

(9) $${\mathrm{CC}}_{s,j}=\frac{\sum_{t=1}^{N_{s,j}}\left(y_{t}-\bar{y}\right)\left({\hat{y}}_{t}-\bar{\hat{y}}\right)}{\sqrt{\sum_{t=1}^{N_{s,j}}{\left(y_{t}-\bar{y}\right)}^{2}}\sqrt{\sum_{t=1}^{N_{s,j}}{\left({\hat{y}}_{t}-\bar{\hat{y}}\right)}^{2}}}$$

The Overall CC was the mean of the valid subject–joint CC values; trajectories from different subjects were not concatenated.

### 3.2. Performance in Walking (WAK) Task

During the walking (WAK) task, RTGNet achieved the best overall prediction accuracy among all evaluated models, with an Overall MAE of 3.87°, RMSE of 5.25°, and $R^{2}$ of 0.81, and CC of 0.88. Compared with the temporal-only baselines, RTGNet reduced the Overall MAE by 21.8% relative to BiLSTM and 24.0% relative to TCN.

Compared with RP-based image models, RTGNet also showed consistent advantages. It reduced the Overall MAE by 12.6% compared with the RP-only model and by 16.8% compared with RP-ResNet50. Among the lightweight modern RP backbones, RP-FastViT achieved the best performance, with an Overall MAE of 4.55° and $R^{2}$ of 0.74; however, it remained inferior to RTGNet.

The detailed joint-specific and overall performance results for the WAK task are summarized in Table 2.

**Figure 6:**
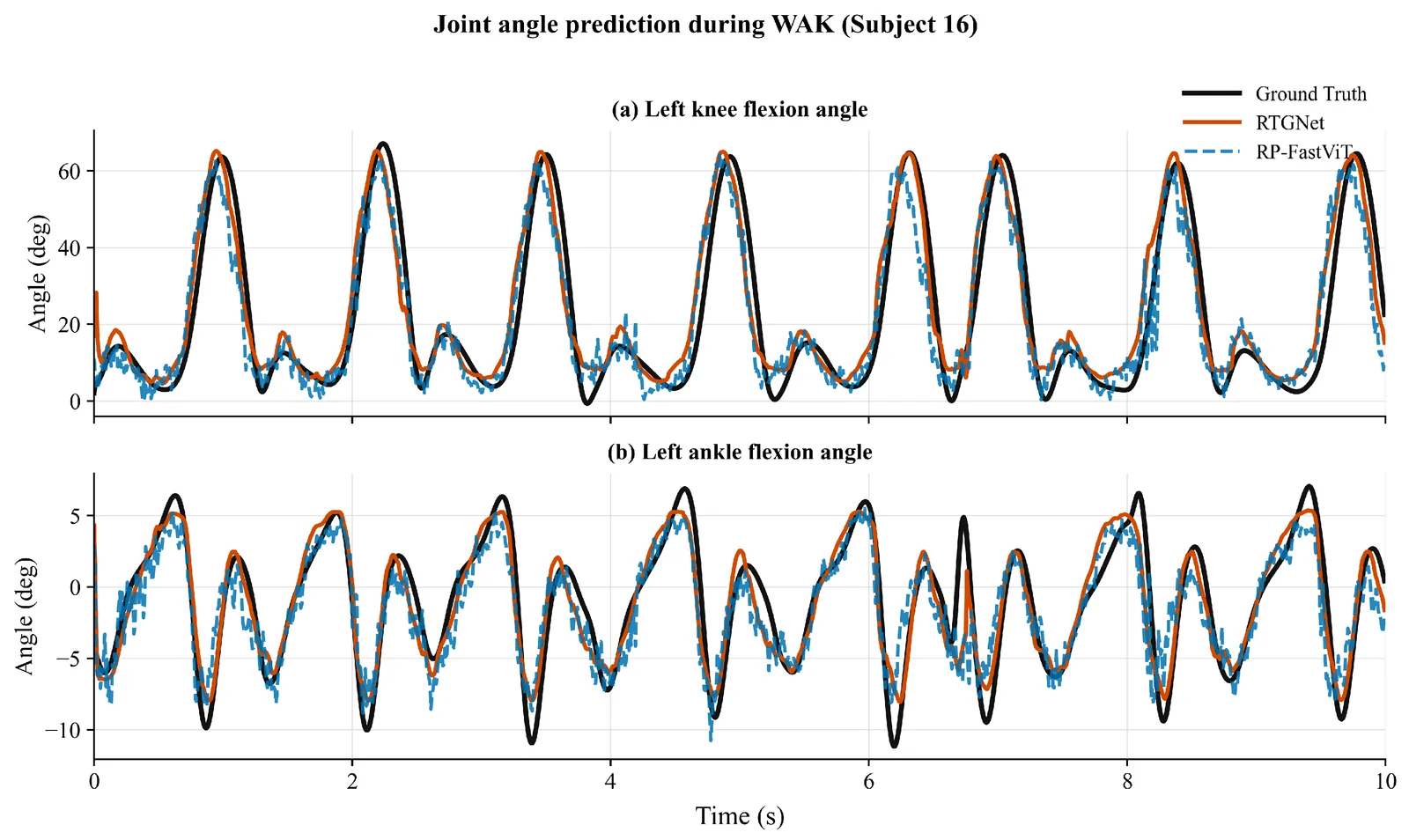
Continuous prediction trajectories of the knee and ankle flexion angles for a representative subject (Subject 16) during the walking (WAK) task.

Figure 6 shows representative predicted and ground-truth trajectories for Subject 16 during the WAK task.

**Figure 7:**
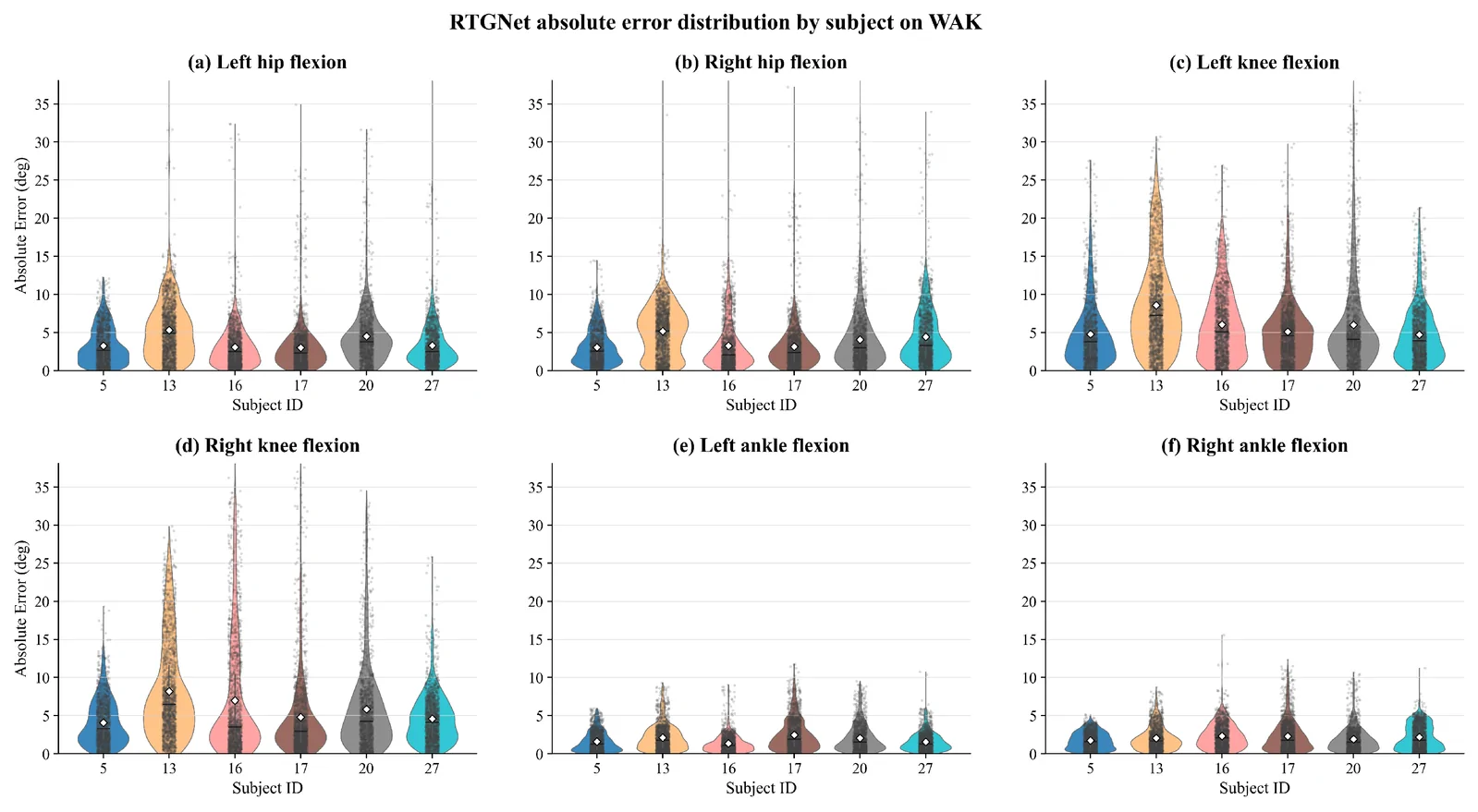
Cross-subject absolute error distributions of RTGNet during the walking (WAK) task. Each violin represents the window-level absolute-error distribution for one test subject, identified by the Subject ID on the x-axis. White diamonds denote mean errors, and the central bars denote medians.

Figure 7 shows the subject-wise absolute-error distributions of RTGNet for the WAK task. Errors were generally lower and more concentrated for the ankle joints, whereas the hip and knee joints showed greater inter-subject variability, including long-tail errors for some participants such as Subject 13.

### 3.3. Performance in Stair Ascent (UPS) Task

**Figure 8:**
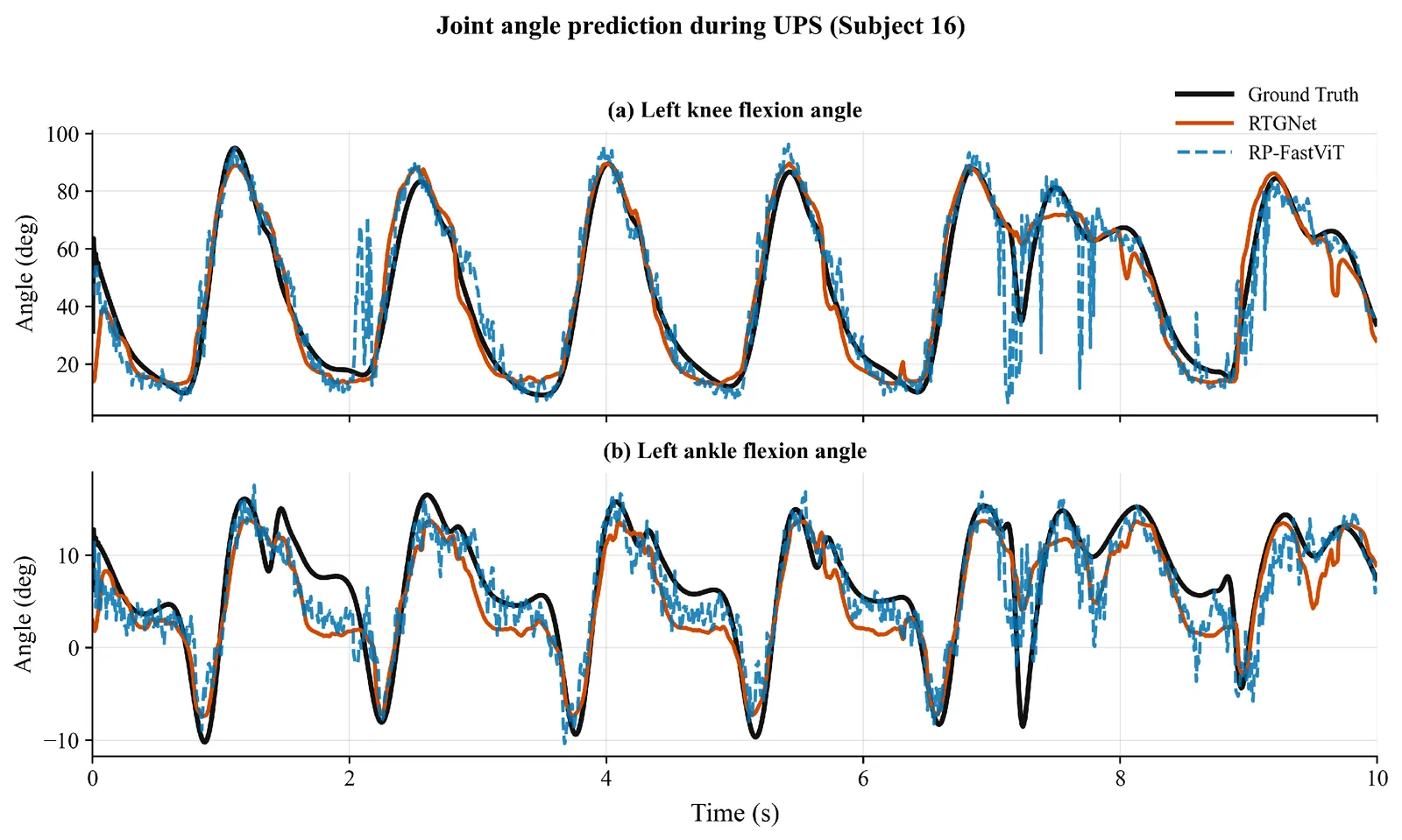
Continuous prediction trajectories of the knee and ankle flexion angles for a representative subject (Subject 16) during the highly dynamic stair ascent (UPS) task.

In the stair ascent (UPS) task, RTGNet maintained the best overall prediction performance despite the increased biomechanical complexity of anti-gravity movement. It achieved an Overall MAE of 4.53°, RMSE of 6.47°, $R^{2}$ of 0.84, and CC of 0.92, outperforming all temporal-only and RP-image-based comparison models. Compared with the temporal baselines, RTGNet reduced the Overall MAE by 7.9% relative to BiLSTM and by 27.6% relative to TCN. Although BiLSTM showed competitive performance in this task, RTGNet still produced lower errors across all six joint angles and achieved a higher $R^{2}$ and CC, indicating stronger robustness in modeling stair-ascent dynamics, as shown in Figure 8.

The advantage of RTGNet was more pronounced when compared with RP-image-only models. Relative to RP-only, RTGNet reduced the Overall MAE by 32.6%. It also outperformed RP-ResNet50 by 37.4% and exceeded recent lightweight RP backbones, including RP-EfficientViT, RP-RepViT, and RP-FastViT. Among these RP-based image models, RP-FastViT and RP-RepViT performed better than RP-ResNet50, but their errors remained substantially higher than those of RTGNet. These results suggest that spatial recurrence textures alone are insufficient for capturing the non-stationary dynamics of stair ascent.

Overall, the UPS results demonstrate that RTGNet effectively integrates recurrence texture representations with temporal dependencies, enabling stable continuous joint angle estimation under more challenging locomotor conditions. The detailed joint-specific and overall performance results for the UPS task are summarized in Table 3.

**Figure 9:**
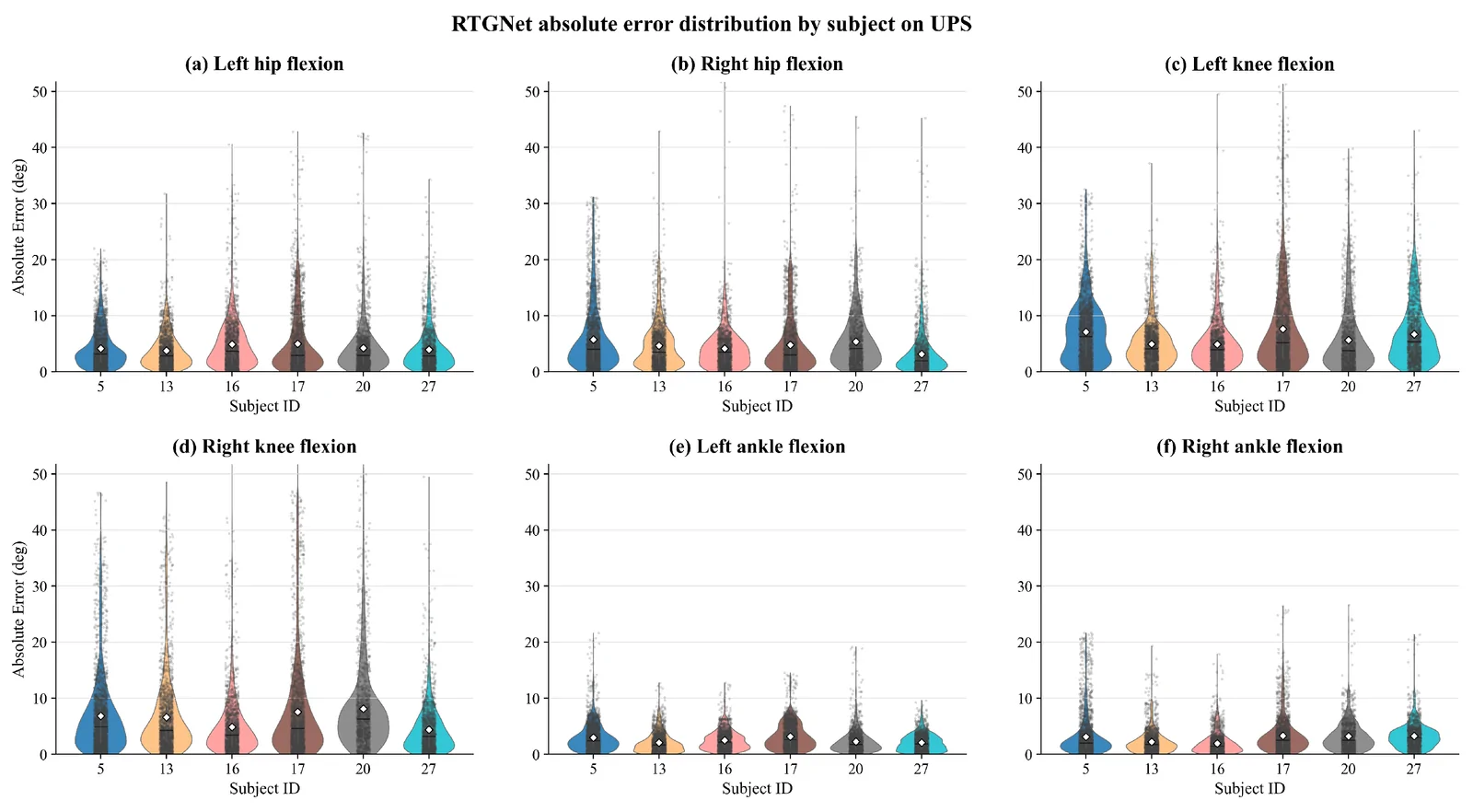
Cross-subject absolute error distributions of RTGNet during the stair ascent (UPS) task. Each violin represents the window-level absolute-error distribution for one test subject, identified by the Subject ID on the x-axis. White diamonds denote mean errors, and the central bars denote medians.

To illustrate the impact of increased task complexity on individual prediction variance, Figure 9 visualizes the subject-wise error distributions for the UPS task. As expected in non-stationary anti-gravity movements, the overall variance is noticeably larger compared to level walking. While the ankle joints still maintain relatively narrow interquartile ranges, proximal joint kinematics appeared more difficult to predict in the present evaluation. This is reflected in greater inter-subject variability and extreme outliers, most notably the prominent long-tail distribution in the right knee of Subject 20. These findings suggest that high-intensity dynamic tasks may amplify biomechanical heterogeneities among subjects; however, the median errors remained within a stable range.

### 3.4. Ablation Study

#### Effect of Model Components

An ablation study was conducted on the WAK task to evaluate the contribution of key modules (Table 4). The full RTGNet (MAE = 3.871°) outperformed all variants. Removing the gated fusion module led to a performance drop (MAE = 4.107°), indicating lower error with adaptive gating than with direct feature concatenation. The exclusion of the CBAM attention module increased the MAE to 4.181°. Notably, utilizing the original loss function instead of the proposed Peak-Enhanced Huber-TopK loss resulted in a substantial increase in MAE (4.467°), supporting the utility of emphasizing high-error samples in the present evaluation.

In the “RTGNet w/o gated fusion” variant, direct concatenation of the RP and temporal embeddings replaced the adaptive gate, while the prediction head was unchanged.

### 3.5. Cross-Branch Representation Analysis

The RP-CNN and BiLSTM embeddings were extracted from the same trained multimodal RTGNet checkpoint for all test windows. The mean absolute Pearson correlation between the two 256-dimensional embedding spaces was 0.057, with a median of 0.045. Linear CKA similarity was 0.095, and the mean sample-wise cosine similarity was 0.182. These results indicate low similarity between the two learned embedding spaces.

**Figure 10:**
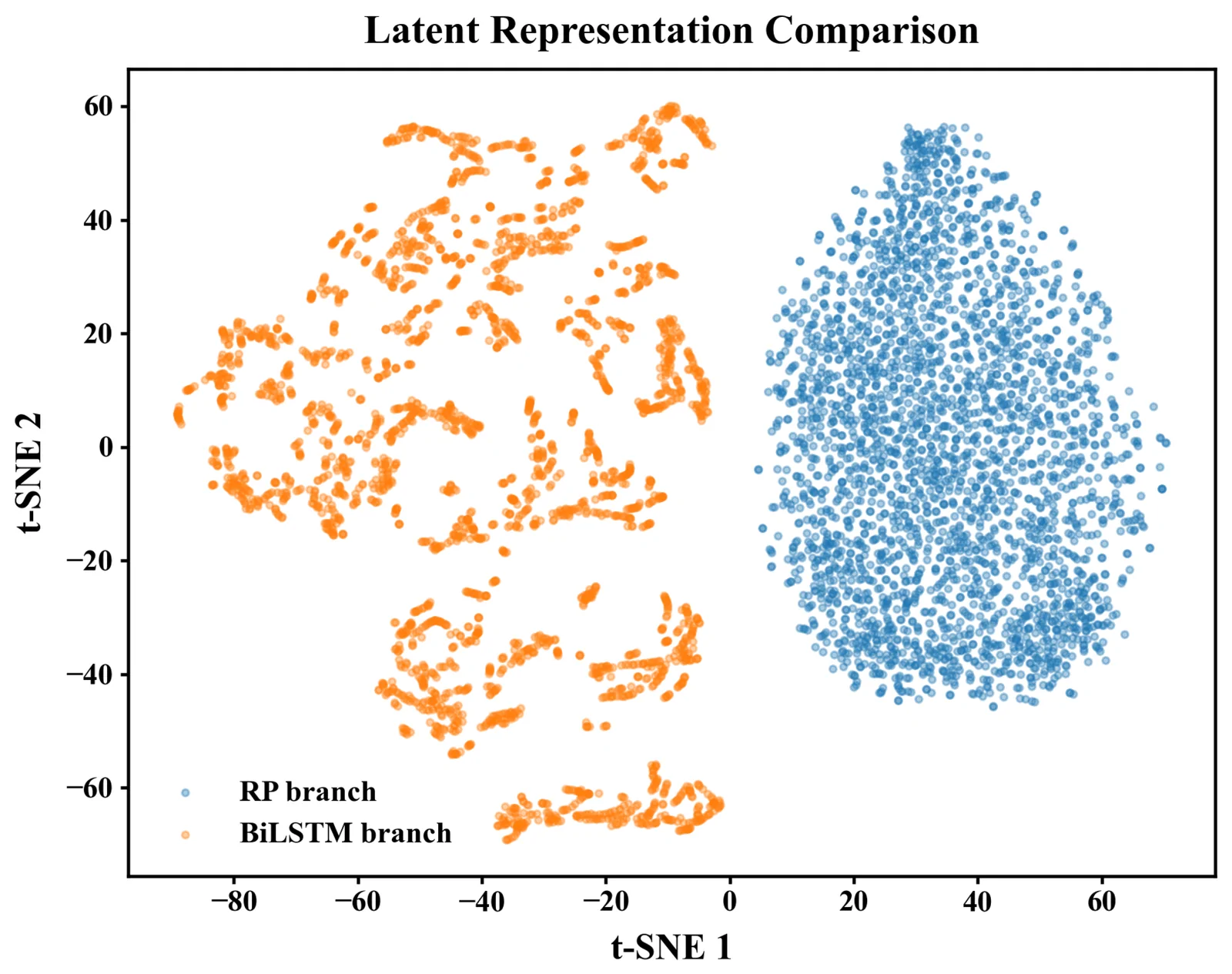
Two-dimensional t-SNE visualization of the 256-dimensional embeddings extracted from the RP-CNN and BiLSTM branches of the trained multimodal RTGNet on the WAK test set. The projected distributions illustrate differences between the learned branch representations.

Figure 10 provides a two-dimensional t-SNE visualization of the two embedding sets. The RP-CNN and BiLSTM embeddings occupied different regions of the projected space. The sample-wise prediction-error correlation between the branches was 0.297. Together with the ablation results, in which the fused model outperformed both single-branch baselines and adaptive gated fusion achieved lower error than direct concatenation, these findings are consistent with partially complementary predictive information.

#### Sensitivity to Window Length

To justify the selection of window-related parameters in RP construction, we further evaluated four temporal window configurations on the 120 Hz training timeline: 40/20, 60/30, 80/40, and 100/50. Here, the two numbers denote the window length and stride used for training and validation, respectively, while the test stride was fixed to 1 for dense evaluation. As summarized in Table 5, the default 60/30 configuration achieved the best overall performance, with an MAE/RMSE/$R^{2}$ of 3.87/5.25/0.81. In comparison, the 40/20 setting yielded slightly inferior but still competitive results, whereas the 80/40 and 100/50 configurations led to further performance degradation. These findings indicate that the prediction performance is influenced by the RP window configuration, and support the use of a 0.5 s temporal context as the most appropriate setting for constructing informative recurrence patterns from sEMG signals.

### 3.6. Computational Complexity and Latency

To assess the accuracy–efficiency trade-off, representative temporal, RP-based, and multimodal models were profiled using their WAK checkpoints on an NVIDIA GeForce RTX 5060 graphics processing unit (GPU). All measurements used batch size one with a sequence input of 1×60×9 and, where applicable, an RP input of 1×9×60×60. After 100 warm-up iterations, latency was measured over 300 forward passes. Floating-point operations (FLOPs) were estimated for one forward pass using THOP. For RP-based models and RTGNet, the RP-processing-and-inference delay includes online RP construction and model inference; it is not applicable to sequence-only models. These measurements exclude signal acquisition, filtering, window accumulation, and host–device transfer.

Table 6 shows that RTGNet requires 3.07 M trainable parameters and 0.423 G FLOPs per window, which is substantially smaller in parameter count than RP-ResNet50 (24.05 M) while retaining the additional temporal branch. Its mean network forward-inference time was 18.13 ms, and its RP-processing-and-inference delay was 21.45 ms. The 95th-percentile RP-processing-and-inference delay of RTGNet was 26.33 ms. BiLSTM and TCN have no RP-processing-and-inference delay because they do not construct or process RP images; their network inference times are reported separately. These results quantify the computational cost associated with the improved prediction accuracy of RTGNet.

## 4. Discussion

In this study, we proposed RTGNet, a dual-branch framework for continuous lower-limb joint angle prediction from multichannel sEMG signals. In the present cross-subject evaluation, jointly modeling RP-based recurrence textures and temporal sequence information yielded lower errors than either single-branch baseline. This result supports the utility of combining structural and temporal cues when modeling the nonlinear relationship between sEMG activity and joint kinematics.

A key observation is that the RP-based spatial branch and the temporal sequence branch learn partly different feature representations. The RP branch describes channel-wise nonlinear recurrence structures, whereas the BiLSTM branch models sequential dependencies and phase-dependent temporal modulation. The lower errors of the fused model relative to the single-branch and direct-concatenation variants are consistent with a benefit from combining these representations through adaptive gated fusion.

The representation analysis provides supporting evidence that the two branches encode partly distinct information. The low cross-branch correlation and CKA similarity alone do not establish complementarity; however, when considered together with the ablation results, they are consistent with partially complementary predictive information. Adaptive gated fusion integrates the two representations at the feature level.

Another important finding is that the peak-enhanced Huber-TopK loss was associated with lower error than the original loss in the present ablation. Conventional regression losses may underweight samples with large instantaneous errors when averaged over a mini-batch. In contrast, the proposed loss increases the contribution of high-error samples within each mini-batch. These samples may include windows around rapid transitions or extreme motion phases, but the current analysis does not isolate peak-specific performance.

The joint-specific error patterns further suggest that prediction difficulty is not uniform across anatomical sites. In both locomotor tasks, ankle angles were generally associated with lower errors, whereas knee angles remained more difficult to estimate. This trend may reflect differences in neuromuscular control complexity across joints. Compared with the ankle, the knee is influenced by more variable multi-muscle coordination and stronger subject-specific differences, which may make its sEMG-to-kinematics mapping harder to generalize in a cross-subject setting. These findings indicate that future improvements may benefit from joint-aware modeling strategies or more explicit biomechanical constraints.

The additional window analysis further supports the choice of RP-related temporal parameters. Performance varied with the RP window configuration, and the default 60/30 setting achieved the best overall results among the tested candidates. This suggests that the quality of the recurrence representation depends on using a temporal context that is sufficiently long to preserve informative structure, yet not so long that redundant or less relevant dynamics are introduced. For the present dataset and task setting, a 0.5 s temporal window appears to provide the most appropriate balance.

In the offline evaluation workflow, RTGNet processes a 60-sample sEMG window (0.5 s at 120 Hz) by constructing its channel-wise RP representation and combining it with the corresponding temporal sequence. The batch-size-one experiment reported in Table 6 quantifies an RP-processing-and-inference delay of 21.45 ± 2.67 ms on a desktop GPU. This latency is reported as a computational-efficiency measure for the evaluated model pipeline; it excludes signal acquisition, filtering, window accumulation, host–device transfer, and embedded-platform execution.

Despite these encouraging results, several limitations should be acknowledged. First, all experiments were conducted on a single public dataset, which may limit generalizability to other recording setups, motion protocols, or electrode placements. Second, the current study focused on healthy participants, and the proposed framework has not yet been validated in patient populations or under pathological gait conditions. Third, the experiments were performed in an offline cross-subject setting, and no real-time closed-loop control evaluation was conducted. The present results therefore establish offline predictive feasibility but do not establish readiness for clinical or robotic closed-loop deployment. Real-time use would require causal filtering, optimization or approximation of online RP construction, hardware-specific latency profiling, and closed-loop stability and safety evaluation. Further work is needed to assess the applicability of the proposed method in control-oriented and real-world deployment scenarios.

Overall, the present study shows that combining recurrence-based texture representation with temporal dependency modeling is an effective strategy for sEMG-based lower-limb joint angle prediction. Future work will focus on extending the method to broader locomotor conditions, improving cross-subject generalization, and validating the framework in more realistic assistive or rehabilitation-oriented settings.

## 5. Conclusions

This study proposed RTGNet, a dual-branch deep learning framework for sEMG-driven lower-limb joint-angle prediction that integrates RP-based spatial texture modeling with BiLSTM-based temporal sequence learning. An adaptive gated fusion mechanism combines the two feature representations, while a peak-enhanced Huber-TopK loss emphasizes samples with large prediction errors. Experiments on the SIAT-LLMD dataset under an offline cross-subject setting show that RTGNet outperformed the evaluated temporal-only and RP-based baselines, achieving MAE/RMSE/$R^{2}$ values of 3.87°/5.25°/0.81 for walking and 4.53°/6.47°/0.84 for stair ascent. The representation analysis and ablation results are consistent with partially complementary predictive information from the two branches and support the utility of gated fusion, CBAM attention, and the proposed loss function.

The current study is limited to offline evaluation on a single dataset comprising healthy adults. Although batch-size-one profiling quantified the computational cost of the evaluated model pipeline, future work will focus on cross-dataset validation, model compression and latency reduction, and extension to pathological gait populations.

Overall, RTGNet provides an effective framework for sEMG-based kinematic estimation and a promising basis for future human–machine interaction, exoskeleton-control, and rehabilitation applications. Further translational validation is required before real-world deployment.

## Figures and Tables

**Table 1:** Validation-set sensitivity analysis of the Peak-Enhanced Huber-TopK loss on the WAK task. MAE and RMSE are reported in the normalized target space.

ρ	$\lambda_{\mathrm{topK}}$	$N_{\mathrm{topK}}$	Validation MAE	Validation RMSE
0.01	1	1	0.1060	0.1479
0.01	3	1	0.1113	0.1568
0.01	5	1	0.1115	0.1541
0.05	1	6	0.0986	0.1336
0.05	3	6	0.0983	0.1367
0.05	5	6	0.1004	0.1403
0.10	1	12	0.0995	0.1382
0.10	3	12	0.1027	0.1490
0.10	5	12	0.0986	0.1393

**Table 2:** Comparison of joint angle prediction performance among different models during the walking (WAK) task. Prediction errors are reported in degrees (°); CC is dimensionless and is calculated subject-wise for each joint before averaging.

Model	Joint-Specific MAE						Overall Performance			
	L-Hip	L-Knee	L-Ankle	R-Hip	R-Knee	R-Ankle	MAE	RMSE	$R^{2}$	CC
BiLSTM	4.98	7.77	2.05	5.21	7.21	2.47	4.95	6.55	0.72	0.83
TCN	5.38	7.68	2.26	5.12	7.92	2.17	5.09	6.61	0.72	0.85
RP-only	4.70	6.46	2.15	4.41	6.59	2.29	4.43	6.08	0.75	0.85
RP-EfficientViT [21]	4.77	6.94	2.31	4.85	7.44	2.37	4.78	6.44	0.72	0.82
RP-RepViT [22]	5.04	7.04	2.49	4.93	7.46	2.44	4.90	6.50	0.71	0.81
RP-FastViT [23]	4.66	6.28	2.31	5.02	6.79	2.22	4.55	6.19	0.74	0.83
RP-ResNet50 [24]	4.60	7.34	2.26	4.58	6.93	2.18	4.65	6.39	0.73	0.82
RTGNet	3.74	5.89	1.86	3.88	5.76	2.09	3.87	5.25	0.81	0.88

**Table 3:** Comparison of joint angle prediction performance among different models during the stair ascent (UPS) task. Prediction errors are reported in degrees (°); CC is dimensionless and is calculated subject-wise for each joint before averaging.

Model	Joint-Specific MAE						Overall Performance			
	L-Hip	L-Knee	L-Ankle	R-Hip	R-Knee	R-Ankle	MAE	RMSE	$R^{2}$	CC
BiLSTM	4.74	6.99	2.96	4.98	6.72	3.11	4.92	7.10	0.81	0.91
TCN	5.74	9.43	3.65	6.23	8.96	3.53	6.26	8.02	0.76	0.88
RP-only	6.52	9.49	3.23	7.29	10.35	3.47	6.72	10.78	0.65	0.83
RP-EfficientViT [21]	7.23	11.00	3.76	8.02	11.50	3.83	7.56	11.64	0.58	0.77
RP-RepViT [22]	6.60	10.13	3.46	7.29	10.53	3.70	6.95	10.58	0.64	0.81
RP-FastViT [23]	6.87	10.33	3.30	7.24	10.33	3.35	6.90	10.85	0.65	0.82
RP-ResNet50 [24]	7.01	10.48	3.64	7.45	11.18	3.68	7.24	11.12	0.62	0.80
RTGNet	4.33	6.26	2.55	4.72	6.46	2.85	4.53	6.47	0.84	0.92

**Table 4:** Ablation study results of the proposed RTGNet and its variants for cross-subject prediction during the WAK task.

Model	MAE (°)	RMSE (°)	$R^{2}$
BiLSTM (Temporal-only)	4.949	6.553	0.720
RP-only (Spatial-only)	4.433	6.084	0.749
RTGNet (Full model)	3.871	5.253	0.805
RTGNet + original loss	4.467	6.088	0.752
RTGNet w/o gated fusion	4.107	5.544	0.797
RTGNet w/o CBAM	4.181	5.630	0.777

**Table 5:** Influence of RP window length and stride on prediction performance.

Configuration	Window Length	Train/Val Stride	Test Stride	MAE (°)	RMSE (°)	$R^{2}$
40/20	40	20	1	4.08	5.48	0.79
60/30	60	30	1	3.87	5.25	0.81
80/40	80	40	1	4.25	5.73	0.77
100/50	100	50	1	4.29	5.78	0.78

**Table 6:** Computational complexity and batch-size-one latency of representative models. Values are mean ± standard deviation over 300 measurements on an NVIDIA GeForce RTX 5060 GPU. RP-processing-and-inference delay applies only to RP-based models and RTGNet and includes online RP construction; it is not applicable to sequence-only models.

Model	Params (M)	FLOPs (G)	Inference Time (ms)	RP-Processing-and-Inference Delay (ms)
BiLSTM	0.57	0.033	0.93 ± 0.22	N/A
TCN	1.06	0.060	2.31 ± 0.66	N/A
RP-only	1.94	0.390	17.75 ± 2.26	18.66 ± 2.47
RP-FastViT	3.46	0.570	19.62 ± 3.02	19.85 ± 2.54
RP-ResNet50	24.05	0.346	12.03 ± 1.55	13.35 ± 2.20
RTGNet	3.07	0.423	18.13 ± 1.76	21.45 ± 2.67

## Data Availability

The data analyzed in this study are available from the public SIAT-LLMD dataset [5].

## Abbreviations

The following abbreviations are used in this manuscript:

sEMG	Surface electromyography
RP	Recurrence plot
CNN	Convolutional neural network
TCN	Temporal convolutional network
RTGNet	Recurrence-Temporal Gated Network
BiLSTM	Bidirectional Long Short-Term Memory
CBAM	Convolutional Block Attention Module
MAE	Mean absolute error
RMSE	Root mean square error
MSE	Mean squared error
CC	Pearson correlation coefficient
CKA	Centered kernel alignment
FMG	Force myography
CSV	Comma-separated values
EEG	Electroencephalography
GPU	Graphics processing unit
FLOPs	Floating-point operations
t-SNE	t-distributed stochastic neighbor embedding
WAK	Walking
UPS	Stair ascent
